# Teamwork and Leadership Under Fire at the Epicenter of the COVID-19 Epidemic in the Bronx

**DOI:** 10.3389/fmed.2021.610100

**Published:** 2021-03-18

**Authors:** Yaron Tomer, Michelle Ng Gong, Marla J. Keller, William Southern, Elizabeth A. Kitsis, Grace R. Kajita, Lauren I. Shapiro, Sunit P. Jariwala, Eric J. Epstein

**Affiliations:** ^1^Department of Medicine, Montefiore Medical Center, Albert Einstein College of Medicine, Bronx, NY, United States; ^2^Divisions of Critical Care Medicine and Pulmonary Medicine, Montefiore Medical Center, Albert Einstein College of Medicine, Bronx, NY, United States; ^3^Division of Infectious Diseases, Montefiore Medical Center, Albert Einstein College of Medicine, Bronx, NY, United States; ^4^Division of Hospital Medicine, Montefiore Medical Center, Albert Einstein College of Medicine, Bronx, NY, United States; ^5^Division of Rheumatology, Montefiore Medical Center, Albert Einstein College of Medicine, Bronx, NY, United States; ^6^Division of General Internal Medicine, Montefiore Medical Center, Albert Einstein College of Medicine, Bronx, NY, United States; ^7^Division of Allergy and Immunology, Montefiore Medical Center, Albert Einstein College of Medicine, Bronx, NY, United States; ^8^Division of Endocrinology, Montefiore Medical Center, Albert Einstein College of Medicine, Bronx, NY, United States

**Keywords:** COVID-19, department of medicine, pandemic, teamwork, leadership, telehealth, coordination, communication

## Abstract

The first Covid-19 patient was admitted to Montefiore Medical Center (MMC) on March 10, 2020. Soon thereafter there was a rapid and exponential surge of Covid-19 admissions to MMC that could have resulted in catastrophic consequences if MMC had been overwhelmed, as happened in Europe. To adjust to this crisis our institution, under the inspiring leadership of Dr. Philip Ozuah, President and CEO of Montefiore Medicine, adopted an “all hands on deck” approach, mobilizing our entire workforce to expand our units to accommodate the growing number of patients being admitted. Given that the internal medicine (IM) and ICU units are part of the department of medicine (DOM), the DOM was at the center of this mobilization. The DOM is the largest department at MMC and mobilizing it required careful planning, seamless teamwork, and strong leadership. To achieve that goal, we applied a framework that we designate the “**3C framework**,” denoting **C**oordination, **C**ommunication, and **C**ollaboration. In this report we describe the many initiatives the Montefiore Einstein DOM implemented during the Covid-19 pandemic using the **3C** framework. These included establishing the Medicine Covid-19 Taskforce to lead our efforts, starting a daily newsletter for up-to-date communications, rapidly expanding the ICU and IM units, converting most specialty inpatient consults to eConsults, coordinating research studies, and more. The goal of this report is to serve as a guide on how the 3C framework helped us organize, mobilize, and energize the department of medicine effectively and efficiently during this unprecedented crisis.

## Introduction

**Figure 1:**
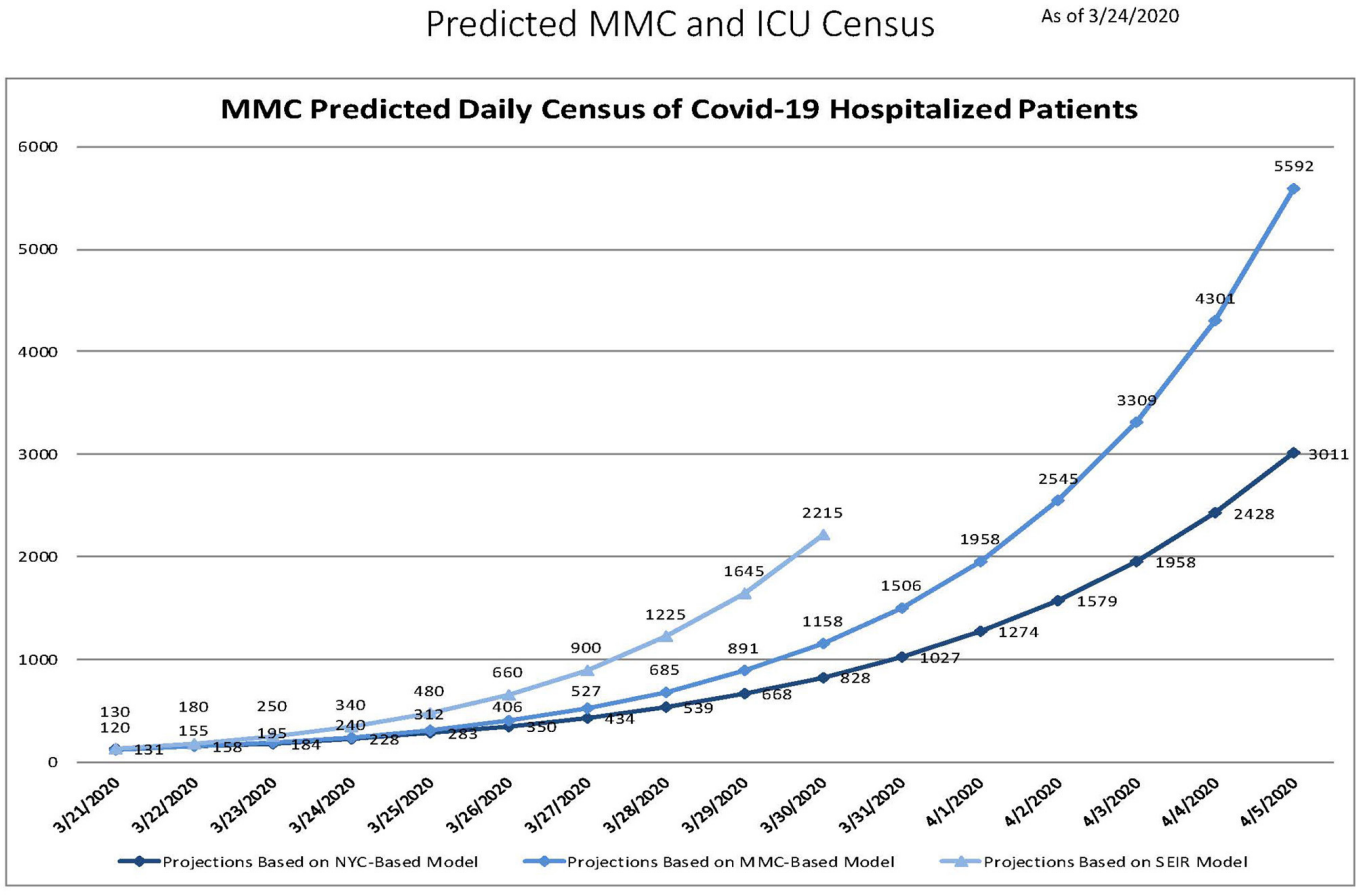
Three predictive models generated on March 24, 2020 showing the expected exponential increase in daily Montefiore Medical Center census over the following 2 weeks.

The first Covid-19 patient was admitted to the Montefiore Medical Center (MMC) intensive care unit (ICU) on March 10, 2020. Within 2 weeks, 345 Covid-19 patients were hospitalized at MMC (comprising 4 hospitals, Montefiore, Weiler, Children's Hospital, and Wakefield). As the rapid surge of Covid-19 admissions to MMC accelerated, predictive models were used to estimate the number of patients likely to be admitted in the coming days, and they all painted a grim picture. Three models (Montefiore Einstein Department of Epidemiology and Population Health model, New York City (NYC) Department of Health model, and Susceptible, Exposed, Infected, Resistant [SEIR] model) predicted that Montefiore would be overwhelmed with Covid-19 patients by early April, even after expanding bed capacity to its limits (Figure 1). This outcome could have resulted in catastrophic consequences, like the flooding of hospitals that was seen in Italy just a few weeks earlier (1, 2), where hospitals exhausted resources and personnel needed to care for the patients. To prevent this bioethical nightmare scenario (3) our institution, adopted an “all hands on deck” approach, mobilizing our entire workforce to expand our units to accommodate the growing number of patients being admitted. Given that the medical and ICU units are part of the department of medicine (DOM), the DOM was at the center of this mobilization.

**Figure 2:**
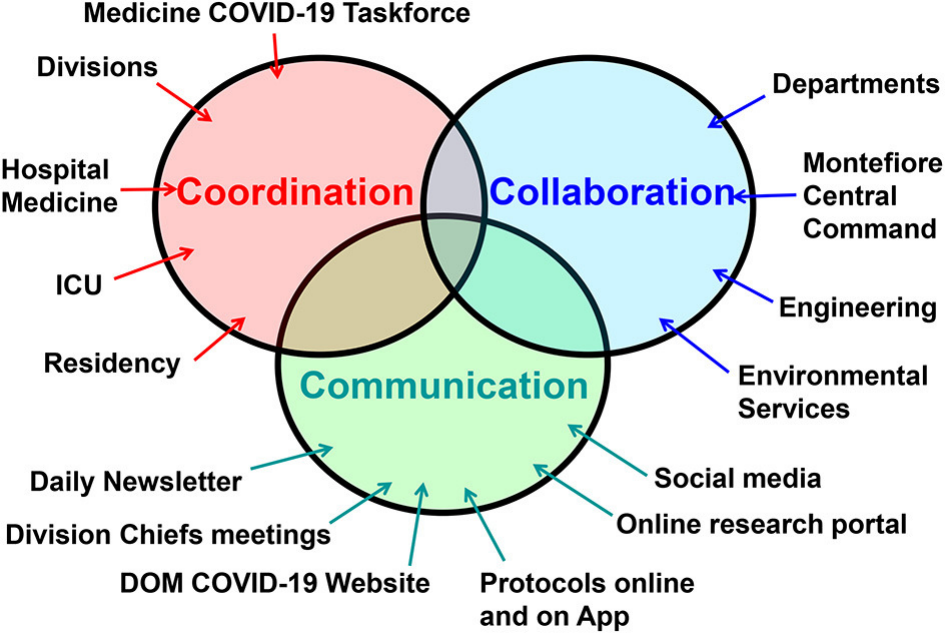
The 3C framework consists of three elements: coordination between the department of medicine (DOM) divisions, ICU, Covid-19 units, residency training program, as well as other arms of the DOM. This was led by the Medicine Covid-19 Taskforce; Communication between all arms of the DOM was achieved via a daily newsletter, division chiefs meetings, online resources, and even social media; Collaboration with all arms of the institution, especially with Montefiore Medical Center administration and central command, was essential to ensure synergy and uniformity of efforts within the entire institution.

With 15 divisions the Montefiore Einstein DOM is the largest department in our institution and mobilizing such a large department required careful planning, seamless teamwork, and strong leadership. To achieve that goal, we applied a framework for our efforts and initiatives during the crisis that we designate the “**3C framework**”, an abbreviation for **C**oordination, **C**ommunication, and **C**ollaboration (Figure 2). In this report we describe the many initiatives the Montefiore Einstein DOM implemented during the Covid-19 pandemic using the **3C** framework. For a summary of our initiatives within the 3C framework see Table 1. The goal of our report is not to summarize the MMC Department of Medicine's experience during Covid-19 (such a summary would be beyond the scope of this manuscript), but to serve as a guide on how the 3C framework helped us organize, mobilize, and energize the department of medicine effectively and efficiently during this unprecedented crisis.

**Table 1:** List of Department of Medicine (DOM)-led initiatives in which the 3C framework was implemented.

**Initiative**	**Description**	**DOM participants**	**Coordination**	**Communication**	**Collaboration**
MCT^*^	A taskforce charged with coordinating all operations of the DOM during the Covid-19 crisis.	The leadership of the DOM (18 members)	Every initiative or plan was reviewed and discussed by the MCT and coordinated by it	All MCT decisions were communicated to the entire DOM through the Daily Newsletter	All MCT initiatives or plans were in cooperation with Montefiore Command Center and with other departments. Different MCT members based on their area of responsibility in charge of collaboration.
ICU expansion	ICU beds expanded from ~ 100 to ~306; additional ~100 patients on ventilators admitted to medicine units	Critical Care and Pulmonary divisions leadership	ICU staffing by residents and fellows was coordinated with PD's and Division chiefs	Deployment decisions were communicated to DC and program directors at DC meetings held twice a week, and directly to physicians deployed	Deployments were in cooperation with the Central Deployment command center of Montefiore and other department chairs
IM units' expansion	Medicine beds expanded from ~700 to ~ 1,100	Hospital medicine division leadership	Medicine unit staffing was coordinated with PD's and Division Chiefs	Deployment decisions were communicated to division chiefs, program directors and physicians deployed	Allied residents (from non-Medicine departments) were deployed in cooperation with other departments and Central Deployment command center.
Creating and disseminating unified Covid-19 treatment protocols	Departmental experts created protocols for treating Covid-19 disseminated to all providers (e.g., steroid, anti-coagulation, DKA, proning, end of life protocols, etc)	Led by the division of Hospital Medicine with participation of specialists with expertise in subject area of individual protocols	All protocols were reviewed by several experts and DOM leadership before being approved. Data supporting protocols were included in the protocol	Protocols are accessible via an online portal, smart phone Application (MedProtocols), and DOM Covid-19 website; Disseminated by text messages to all providers, in daily Newsletter, and through a nightly conference calls with all providers	Collaboration with legal department to add appropriate disclaimers; cooperation with other departments and dissemination to all departments participating in the care of Covid-19 patients
Inpatient eConsults	Converted the majority of inpatient consults to eConsults	DOM director of faculty practice, DOM QI co-director, DOM director of innovation, hospital medicine leadership	Director of DOM faculty practice coordinated all involved in development and implementation of inpatient eConsults	Daily Newsletter and at DC meetings	Creating of eConsults required collaboration with the EPIC team and all other departments since all departments participated
Tele-visits for outpatient	Converted nearly all outpatient visits to tele-visits	Project led by DOM faculty practice leadership and administration	Director of DOM faculty practice coordinated the efforts	Daily Newsletter and at DC meetings	DOM practice leadership rolled out tele-visits in cooperation with Montefiore FPG group, EPIC team and other departments
Education: Covid-19 training and education	Educational activities included: **(1)** Medicine Grand Rounds series on Covid-19 (via Zoom); **(2)** Production of 5 video lectures on treating Covid-19 patients with ARDS on a vent;	**(1)** Grand Rounds organized by VC for faculty affairs; speakers included DOM faculty & outside speakers; (2) Videos lectures given by Pulmonary & Critical Care faculty	**(1)** Grand Rounds were coordinated by Chair, VC for faculty affairs, and administration; **(2)** Videos production coordinated by DOM director of media and communications and Einstein department of communications	Grand Rounds and videos were communicated by special announcements and via Daily Newsletter	Grand Rounds organized in cooperation with other departments (e.g. Psychiatry, Family & Social Medicine) to include their experts as speakers; coordinated to have members of all departments and Einstein researchers attend the Zoom lectures
Research: Clinical Trials/Studies	Within < 2 weeks of the first Covid-19 patient admitted to Montefiore the DOM began participating in clinical trials. Altogether the DOM participated in 8 randomized clinical trials: 3 Remdesivir; 1 Sarilumab; 2 Leronlimab; 1 Convalescent Plasma; 1 HCQ; and a CDC surveillance of HCW	Pulmonary & CCM divisions, ID division, HM division, all deployed DOM physicians (participated in recruiting patients)	Trials vetted by VC for research (MK) and chair of Medicine (YT) and coordinated among Divisions and PI's	Communication about trials, inclusion/exclusion criteria disseminated via Daily Newsletter and DOM Covid-19 Website	All clinical trials performed in cooperation with EPIC team and other participating departments (e.g. surgery)
Research: Retrospective analysis of Montefiore Covid-19 data	About 200 retrospective analyses of Montefiore Covid-19 data performed by DOM faculty	Faculty from all DOM divisions	Established a committee consisting of 10 division chiefs to review all research projects in the DOM to achieve the following goals: (1) have large and diverse collaborative groups for each project; (2) have appropriate expertise for each project; (3) have the proper tools and study design to achieve aims(4) ensure no overlap or duplication between projects; (4) engage junior faculty and trainees in projects.	All projects were uploaded to an online portal so they can be viewed by all DOM faculty, DC were updated at our meetings on progress in review and approval of projects by the committee	Cooperated with other departments on some projects (e.g. department of epidemiology and population health).
Innovation	Implemented innovative strategies to treat Covid-19 patients: **(1)** Cardiac telemetry patch; **(2)** CGM for Covid-19 inpatients; **(3)** Cellavision autoanalyzer for hematology; **(4)** IV lines and CRRT tubing outside patients' rooms in ICU's (**5)** Critical Care Command Center to provide 24/7 expertise, remote monitoring, and resource allocation to critically ill patients across all campuses (**6**) Portable bedside monitors with remote link to Critical Care Command Center to turn any hospital bed into a temporary high intensity monitored setting (**7**) Intubation drapes and boxes to minimize aerosolization of respiratory secretions during emergent intubations (**8**) Proning teams	Faculty from all divisions	Activities coordinated by MCT	Daily Newsletter	Collaboration with Montefiore Command Center and EPIC team as needed
Alumni volunteers	~40 alumni physicians volunteered to come to Montefiore and help. Of them 9 were deployed.	Deployment of alumni efforts were led by VC for faculty affairs	Efforts coordinated by MCT	Daily Newsletter	Cooperated with Montefiore Command Center for logistics, Care Management Organization for credentialing, EPIC team for EPIC access
Donations	Using social medial and our Newsletter put out a call for donations both monetary and non-monetary (e.g., PPE, food, scrubs, pulse oximeters, and more).	Efforts to secure donations were led by the DOM senior administrator and her staff	Efforts coordinated by MCT	Daily Newsletter and social media	Collaboration with department of finance for establishing funds to hold monetary donations, and with procurement department for donations of supplies
Community outreach	Launched a dynamic social media campaign calling for submissions of notes, photos, and videos from children (and adults) in our community supporting Montefiore healthcare heroes.	Efforts led by the DOM director of media and communications	Efforts coordinated by MCT	Daily Newsletter and social media	Cooperated with Montefiore marketing to expand campaign and produce and disseminate videos featuring submissions received from members of our community
Improving morale	Launched a large campaign dubbed #MontefioreProud on social media and at Montefiore. #MontefioreProud T-shirts, bumper magnets and buttons were distributed widely to HCW	Campaign led by DOM chair and senior administrator and their staff	Efforts coordinated by MCT	Daily Newsletter and social media	Cooperated with Montefiore marketing to produce and disseminate marketing materials and to promote #MontefioreProud on social media

* *MCT, Medicine Covid-19 Taskforce; ICU, intensive care unit; DOM, department of medicine; ID, infectious diseases, HM, hospital medicine; PD, program director; HCW, healthcare workers; DC, division chiefs; FPG, faculty practice group; PI, principal investigator; HCQ, hydroxychloroquine; CGM, continuous glucose monitors; VC, vice chair*.

## Background: The Department of Medicine at Montefiore Medical Center

The MHS includes 11 hospitals located in the Bronx, Westchester County, and the Hudson Valley in New York State. MMC comprises four MHS hospitals located in the Bronx and includes the Montefiore Hospital, Weiler Hospital, Children's Hospital at Montefiore, and Wakefield Hospital, which together have approximately 1,500 beds including 108 intensive care unit (ICU) and 30 intermediate care unit beds, 93,000 annual admissions, and more than 200 outpatient care sites. The current manuscript focuses on the implementation of the 3C framework by the DOM at the MMC.

The DOM is the largest department at MMC and includes 25 inpatient units, 3 medical and cardiac ICUs, and 754 beds, with approximately 47,000 discharges per year. The DOM comprises 15 divisions and has 1,293 providers (of them > 800 full time faculty). The DOM trains 429 residents and fellows in 3 residencies (Moses-Weiler IM residency training 168 residents, Wakefield IM residency training 77 residents, and Dermatology residency training 19 residents) and 15 fellowships (training a total of 165 fellows). Of note the Critical Care Division includes all 7 ICU's and 2 step-down units including the surgical ICU's.

The DOM is also the largest research department at Einstein and Montefiore with approximately $96 million in total grant money received, 150 grants, and 140 ongoing clinical trials (pre-Covid-19) in 2019.

## The Challenges Faced by the MMC Department of Medicine

Challenges faced by the Department of Medicine included: the need to rapidly deploy physician staffing to care for a rapidly expanding inpatient census, provide support for physicians newly deployed to the inpatient setting as well as non-IM physicians who were deployed, stay abreast of the rapidly expanding body of research on Covid-19 to develop and continuously update standardized care protocols, develop processes for the triage and appropriate care of patients being placed in expanded ICUs and non-traditional care spaces, develop and rapidly initiate Covid-19-related research, maintain real-time communication with all providers, and offer emotional and logistical support to physicians and physician assistants (PAs) who were delivering care in extremely challenging and distressing conditions. Residents were the primary providers of inpatient care during the epidemic. In addition to postponing their usual educational activities, all residents were also exposed to the unpredictability and grief associated with caring for Covid-19 patients, during a formative period in their careers.

## Coordination, Communication, and Collaboration

### Medicine Covid-19 Taskforce: The Key to Coordinating the DOM Efforts

To coordinate the deployment and activities of such a large workforce we established a unique task force to centralize and synchronize all DOM activities during the Covid-19 crisis. This task force, the **M**edicine **C**ovid-19 **T**askforce (**MCT**), was charged with seamless coordination and implementation of all initiatives and operations of the DOM. The MCT included 18 members representing all arms of the DOM (Table 2), and it convened every morning through a conference call. During these structured conference calls, every DOM leader and member of the MCT gave a detailed report updating all members on activities, challenges, and plans within their domain. At the end of each presentation we discussed what coordination and synchronization of efforts and resources were needed to achieve our goals and implement our plans and how to communicate these to the entire department. The most important coordination of efforts involved deployment of faculty and trainees. The regular updates and discussions at the MCT ensured that all new ICU and medicine units that were being built and opened overnight were always properly staffed with attendings and trainees.

**Table 2:** Members of the medicine covid-19 taskforce and their roles in coordinating DOM efforts and initiatives.

**Role in the DOM**	**Charge during Covid-19**	**Comments**
Chair	Leading all IM departmental efforts and activities	Chaired the MCT
Chief of Pulmonary & Critical Care Divisions	Leading ICU expansion, ICU operations, protocols, staffing, ventilator management and protocols, critical care resource allocation, emergency responses, supervising all clinical trials performed in the ICU's	Either the Chief or her designee participate in all MCT meetings
Chief of the Division of Hospital Medicine	Leading Medicine Units expansion, Medicine Units operations, protocols, staffing, supervising allied (non-medicine) providers working on medicine or medicine-converted units, supervising all clinical trials performed in the medicine units	
Chief of the Division of Infectious Diseases (ID)^*^ and Member of the Infection Control Team (ICT)	Leading all activities of the ID division operations and staffing, working with Hospital Epidemiologist and ICT on ID protocols for the hospital, leading major studies such as the Convalescent Plasma study	
Residency Program Directors for Moses-Weiler and Wakefield campuses	Leading and supervising all the residents including managing their deployment, quarantine, Covid-19 infections, protocols, as well as training and education during the Covid-19 crisis	Montefiore has 2 residency programs with a combined 243 residents
Vice Chair for Faculty Affairs	Overseeing faculty credentialing, deployment of alumni volunteering to work at Montefiore, communicating departmental updates to all faculty	About 40 alumni volunteered to come and be deployed at Montefiore
Vice Chair for Research	Overseeing all clinical trials in the department, vetting all proposals for clinical trials presented to the department to ensure adequate infrastructure and rapid implementation, overseeing the shutting down and re-opening of all wet-bench labs and clinical research, overseeing all retrospective analyses of Covid-19 data, overseeing all IRB submissions	Montefiore had numerous requests for participation in clinical trials, mostly from pharmaceutical companies, and numerous human studies requiring IRB approval (both clinical trials and retrospective analyses) during the Covid-19 crisis
Associate Chair for Undergraduate Medical Education	Overseeing all medical students Internal Medicine teaching during the Covid-19 deployment, all done remotely using virtual learning platforms	All medical students teaching continued during the Covid-19 epidemic including virtual teaching of Internal Medicine to third and fourth year students that were not allowed to continue bedside learning
DOM Senior administrator, Associate Chair and Director of the Faculty Practice and the Faculty Practice team (administrator, and nursing supervisor)	Overseeing and leading the conversion of all our outpatient activities into tele-health, overseeing all DOM outpatient activities, deploying DOM staff to Covid-19 testing sites and to the frontlines, converting most inpatient consults into e-Consults, overseeing requests and receipts of donations to the DOM	The DOM converted all outpatient visits into tele-visits within 3 days of shutting down our practices, most inpatient consults were also converted into eConsults within a week of the surge. The DOM received numerous donations including monetary donations, PPE, essential supplies such as pulse oximeters and WiFi tablets (to connect patients on the floors with their families), and food.
Associate Chair and Director of Innovation	Overseeing and leading all activities implementing new technology such as tele-health, eConsults, WiFi tablets and EPIC builds for tele-medicine	
Director of Media and Communications	Assisting the Chair with all communications to the DOM, including publishing a Daily Newsletter, and sending messages and information by e-mail and on Social Media	Our Social Media campaign was instrumental for engaging our community to support the DOM frontline health care workers, and in soliciting donations of PPE and other essential supplies.

* *MCT, medicine covid-19 taskforce; IM, internal medicine; ID, infectious diseases; ICT, infection control team*.

Another example in which coordination was important was the development and dissemination of Covid-19 treatment protocols. We were able not only to ensure that all Covid-19 patients admitted to MMC were treated consistently, but also to implement new protocols very rapidly and in many cases much earlier than at other Medical Centers. Moreover, our uniform protocols allowed us to study in real time the impact of our treatment protocols on outcomes. As an example, analysis of Montefiore patient data showed that treatment with steroids was associated with reduced mortality only when the C-reactive protein (CRP) levels were >20 mg/dl, but when CRP was lower than 10 mg/dL steroid treatment was associated with increased mortality (4). This study that was performed in real time contributed to a modification of the protocol to incorporate these new data.

Our coordinated efforts through the MCT enabled us to coordinate many other initiatives. To name a few: (1) The production of critical educational video lectures to train physicians not usually working in the ICU setting; (2) Solicitation, receipt, and distribution of donated personal protective equipment (PPE), and other essential supplies such as pulse oximeters and electronic tablets; (3) Teaching of the medical students; (4) Continuation of essential procedures; (5) Conversion of most activities to telehealth and more.

The key to the 3C framework success was that it operated at all levels of the DOM, not only at the leadership level, that is, within the divisions, as well as at each Covid-19 unit. To facilitate the implementation of the 3C framework within the divisions, the MCT met with all division chiefs twice a week to update them on DOM deployment and other efforts. This ensured that all departmental and divisional efforts were coordinated, that there was no duplication of efforts, and, most importantly, that every issue was immediately addressed in real time. Division Chiefs then held conference calls with their faculty coordinating and communicating with them and receiving communication from their faculty and feedback that was then communicated back to the MCT. To ensure that the 3C framework reached the individual Covid-19 units the chiefs of the Critical Care (MNG) and Hospital Medicine (WS) divisions held regular virtual meetings with the attendings in the ICU's and Covid-19 IM units. Thus, the 3C framework was successful because it was delegated from the DOM leadership to Divisional leadership and to attending physicians at the Covid-19 unit level, and because it was bi-directional.

The chair of medicine (YT) and chiefs of Critical Care Medicine (MNG) and Hospital Medicine (WS) also held regular meetings with hospital administration, nursing leadership, and other departments to ensure seamless collaboration.

### The DOM Covid-19 Daily Newsletter: The Key to our Communication

Transparency is essential for ensuring that members of the department have full trust in departmental leadership and are willing to do whatever is requested of them. This is especially true in times of crisis. Early on the decision was made that with very few exceptions everyone in the department would know what the leadership knew. Therefore, we created a DOM Covid-19 Daily Update, an electronic newsletter that was sent to all members of the department which updated them on everything that was happening—from our hospital Covid-19 statistics, to new protocols, to new relevant publications, and even to discounts by companies to healthcare workers. To ensure that the content was relevant we monitored the number of views of different items and focused on popular items. In addition, we gave the readers an e-mail address to offer suggestions, provide comments, or ask questions. The newsletter was highly successful; it was distributed to > 6,500 people with a daily open rate of up to 65%.

### ICU Expansion

During the Covid-19 crisis our ICU beds at MMC expanded from about 100 ICU beds to 306. This rapid expansion necessitated coordination of deployment between the ICU leadership, residency directors, division chiefs, other departments, and the Montefiore Command Center (which oversaw all ICU deployments). Critical Care not only expanded its ICU bed capacity but also responded to cardiac arrests and rapid emergency calls that increased 5-fold (from an average of 7–8 per day pre-Covid-19 to 33 per day). The Chief of the Critical Care Division (MNG) led and supervised the ICU expansion. The expansion followed specific steps that included: **(1)** Identification of suitable space for an ICU that had enough room for patients in isolation, on ventilators, and with bedside monitoring (e.g., OR recovery unit); **(2)** Coordination with engineering to convert each unit into an ICU, including construction, terminal cleaning, and procuring equipment, especially ventilators; **(3)** Coordination with DOM leadership as well as other department leaders to organize deployment of faculty to the new units; **(4)** Coordination with the program directors and Montefiore Command Center to organize deployment of residents and fellows to the new units; **(5)** Coordination with nursing for the deployment of nurses to the new units; **(6)** Communication to the Emergency Department, Hospital Medicine division, and all providers about the establishment of a new ICU and which patients could be admitted or transferred to the new ICU; **(7)** Development of a Critical Care Command Center to provide 24/7 critical care expertise, remote monitoring of expanded ICU's, deployment of emergency personnel and equipment, point of care ultrasounds, and coordination of transfers of patients, resources and personnel between ICUs and between MMC campuses, and from outside hospitals; **(8)** Development of protocols and procedures for emergency responses like intubation and cardiopulmonary resuscitation to minimize aerosolization and infection risk during the pandemic; **(9)** Development of infection control measures in coordination with respiratory therapy and hospital epidemiology for high risk respiratory procedures in the hospital.

### Expansion of Internal Medicine Units

At the same time as the ICU's were expanding the Internal Medicine (IM) general units were rapidly expanding, too, from approximately 700 beds to ~ 1,100 beds. To achieve this, space for the increased number of patients needed to be identified, and increased physician staffing needed to be mobilized. To provide the needed space, the Covid/Medicine service first expanded into virtually all inpatient spaces that do not traditionally accommodate Medicine patients including Neurology, Oncology and Surgical inpatient units. Next, many non-clinical spaces were rapidly renovated to accommodate inpatients including a large meeting space, unit day rooms, a cafeteria, and rehab gymnasiums. Finally, a large free-standing outpatient surgical center was converted into inpatient units. To provide physician staffing for the expanded service the DOM rapidly constructed novel 3-person inpatient care teams consisting of a Medicine attending, a Medicine resident and an “allied” (non-Medicine) resident. These teams could be constructed and deployed within 24 h of notification of a new staffing need. In addition, several non-Medicine services took over the care of Covid-19 patients including Neurology, Oncology and Pediatrics, and Family Medicine significantly increased their inpatient capacity.

In order to achieve this enormous expansion in a coordinated and organized way the Chief of the Division of Hospital Medicine (WS) implemented several key measures: **(1) Leadership** – with the exponential growth of the general medicine units a new divisional leadership structure was created appointing faculty to new leadership positions, such as director of the IM units at our outpatient facility; **(2) Standards of care** – Divisional leadership in consultation with specialists in different areas such as diabetes and coagulopathies, created uniform protocols for the treatment of Covid-19 patients, including an EPIC Covid-19 admission order set and note template; **(3) Mentoring** – several non-IM units (e.g., pediatrics, neurology) were converted into IM Covid-19 units that were staffed by the original department. Since the providers on these units did not have experience in adult hospital medicine and Covid-19 the Hospital Medicine division provided mentors that came daily and rounded with the providers on these units to help manage their service; **(4) Triaging** – the Division of Hospital Medicine developed criteria for transferring patients from acute Covid-19 units to post- acute areas (non-clinical areas converted into Covid-19 units). **(5) Deployment of physician assistants (PAs)** – PAs were essential on the IM Covid-19 units that did not have residents and the PA leadership at the Hospital Medicine division managed their deployment and training to take on new roles such as being on the code team.

### Medicine Sub-specialty Inpatient Consults

The inpatient subspecialty consultation services had to resolve two main challenges: **(1)** Most consultants were deployed to ICU's or IM Covid-19 units and could not do inpatient consults; and **(2)** PPE was in short supply and consultants coming to do in-person consults would use a large amount of PPE. To resolve both issues the MCT initiated an eConsult service for all inpatient consultations except for a few that required procedures (Table 1).

### Covid-19 Treatment Protocols and Education

One of the challenges our expansion created was that numerous providers with limited ICU or IM experience were deployed to ICU's and IM Covid-19 units. In order to ensure that they could perform their new roles well, we implemented two important initiatives: development and dissemination of detailed and uniform Covid-19 treatment protocols, and production and dissemination of educational Covid-19 video lectures. The Covid-19 treatment protocols were developed by experts in their fields together with the Hospital Medicine Division leadership and were based on the best available data. This approach resulted in very early adoption at Montefiore of life-saving Covid-19 protocols. To mention a few, the Montefiore steroid treatment protocol was rolled out and uniformly implemented on April 6 (4); our anti-coagulation protocol was rolled out on April 5; and the Montefiore subcutaneous (SQ) insulin diabetic ketoacidosis (DKA) protocol was rolled out on April 1. Some of these protocols were disseminated across the country (e.g., our DKA protocol was posted on the American Diabetes Association website and published in UpToDate) (5).

These protocols were easily accessible through an online portal and later through a free downloadable mobile application (MedProtocols, see below). In addition to making the protocols easily accessible from anywhere, any new protocols or modifications to existing protocols were communicated to every provider on the IM units through our daily newsletter, text messaging, and nightly briefs with the Chief of the division of Hospital Medicine. In addition, we converted our weekly Medicine Grand rounds to a virtual format, and held a special Covid-19 series that focused on Internal Medicine subspecialty manifestations of Covid-19.

### Outpatient Deployment and Conversion to Telemedicine

Within 10 days of the first Covid-19 patient being admitted to MMC all but one outpatient practice were closed and many of their staff and providers deployed to Covid-19 units. However, MMC was able to continue most outpatient operations through a rapid conversion to telemedicine. The DOM implemented video visit platforms, and patients were called and instructed that their appointments would be done remotely. One of the challenges we faced in the Bronx was that many patients did not have the ability to do video visits (e.g., they did not have WiFi at home) and therefore only telephone visits were possible for them. The conversion of our outpatient practices to televisits required coordination and collaboration with the Faculty Practice Group, our call center, our Electronic Medical Record team, and our billing services in order to support billing for televisits. To ensure a smooth transition to televisits a DOM team trained and helped providers performing televisits.

### Covid-19 Post-discharge Televisit Program

As large numbers of recovering Covid-19 patients began to be discharged home from Montefiore IM units, there was a need for follow-up care. Therefore, the DOM established a post-discharge follow-up program. Since most of our physicians were deployed we enlisted third year medical students to help with the program which included follow-up phone calls/video visits by physicians or medical students to address the following: **(1)** Ensure that patients were recovering; **(2)** For patients discharged home with pulse oximeters the oxygen saturation readings were evaluated; **(3)** If patients needed follow-up care with specialists, appointments were coordinated and scheduled; **(4)** Patients with financial and socio-economic challenges were assigned a care coordinator from the Montefiore Care Management Organization (the Montefiore accountable care organization) who contacted them to assist. These activities were coordinated with the Montefiore Primary Care group.

### Infection Control and Prevention

Infection control procedures implemented at our institution included universal masking of patients and health care workers (6), appropriate PPE usage when caring for COVID-19 patients, conversion of in-person consults to telemedicine, universal PCR testing upon admission to the hospital, environmental disinfection, and education of staff *via* weekly lectures and the DOM newsletter. When we identified a suspected exposure, we activated an infection control team that performed the contact tracing and implemented isolation and quarantine measures (7). However, the DOM's infection control practices evolved over time in several ways: iterative approaches to PPE distribution were employed until all providers had easy access, and the infection control protocols were adjusted following changes in the CDC recommendations. To implement and disseminate these frequent protocol changes we used our 3C approach by coordinating the implementation of these changes through the MCT and communicating these changes to all providers through our various communication methods (see above). As a result of these efforts, resident sick calls returned to pre-COVID levels within weeks despite an ongoing rise in the number of COVID-19 patients hospitalized at our institution (8).

### Mental Health Support

To address the stress and anxiety that the COVID-19 pandemic induced in healthcare workers the Psychiatry Department at Montefiore established several interventions. These included a support phone line, staff support centers, team support sessions, and more. For a review of these interventions see (9). An additional unique resource - the Montefiore Emotional Support Allies (MESA) – was also established, in which health care workers were individually assigned to a mental health professional (psychologist, psychiatrist, or social worker) who *actively* reached out to them and offered to help. The mental health professionals provided peer support and served as navigators to other emotional health resources. About 20% of those contacted opted for one or more peer support contacts; many finding them meaningfully helpful (Alpert, personal communication).”

### Coordination of Research Studies

From the onset of the epidemic in NYC, clinical and laboratory investigator-initiated research studies aimed to better our understanding of Covid-19 and to improve the care of Covid-19 patients.

#### Clinical Trials

Performing clinical trials during an epidemic when an institution is overwhelmed with patients is challenging. Nevertheless, we decided that it was imperative that we participate in clinical trials aimed at finding new therapies for Covid-19. Our leadership received numerous requests for participation in clinical trials examining new treatments for Covid-19, and those had to be vetted by experts in the field. All clinical trial efforts were centralized and coordinated by the vice chair for research (MJK). In addition, the Albert Einstein College of Medicine established a committee of experts in different relevant fields that evaluated proposed trials to select those that were suitable for our institution. The criteria used to select trials that our institution joined were: **(1)** Convincing premise and rationale for the proposed study; **(2)** Rigorous prior research and preliminary data showing potential efficacy; **(3)** Feasibility of performing the study at Montefiore; **(4)** Adequate infrastructure and staff for rapid implementation. To meet the sheer demands of the pandemic and the urgency of launching new studies quickly, experienced clinical trials coordinators were supplemented with coordinators who volunteered their time when their outpatient studies were suspended. Data collection and entry were assigned to coordinators and volunteers who were based outside of the hospital because of health or infection control restrictions to allow for hospital-based coordinators to concentrate on enrollment and study procedures. With these criteria and strategies, we participated in eight randomized clinical trials during the pandemic: three Adaptive Covid-19 Treatment Trials (ACTT) of remdesivir; sarilumab trial; two leronlimab trials; convalescent plasma trial; an NHLBI-funded hydroxychloroquine trial; and a CDC surveillance study of healthcare workers. These studies helped establish the efficacy of remdesivir, the safety of convalescent plasma, and the lack of efficacy of hydroxychloroquine and sarilumab. The leronlimab trials are ongoing.

#### Retrospective Analysis of Our Covid-19 Cohort

More than 8,000 Covid-19 patients were admitted to MMC and the data collected during their hospitalization was critical to learning about Covid-19. Therefore, investigators and clinicians in the DOM designed protocols and obtained IRB approval for analyzing data collected from all Covid-19 patients cared for at MMC. As these research efforts intensified, it became clear that there was a need for a centralized review and approval process. A large number of retrospective studies were being done simultaneously by various groups within the DOM and it was possible that many of them could overlap. Therefore, we established a **R**esearch **C**ommittee on **C**ovid-19 (RCC) to coordinate all retrospective research efforts and to evaluate each study for premise, importance, feasibility, appropriate expertise, and overlap with other studies. The RCC evaluated approximately 200 projects. To assist the RCC in its work we built an online portal to which every new project was uploaded providing all the necessary information for the RCC to evaluate the project. At the time of the writing of this manuscript, 23 research studies from the DOM at Montefiore have been published. They include studies that had a huge impact on our understanding of Covid-19 (10–16) and on treatment protocols for Covid-19 (4).

#### Other Studies

Non-Covid-related clinical research continued remotely and transitioned to video visits when possible. Some projects used electronic consent (e-consent) to facilitate continued recruitment into studies. DOM investigators submitted 35 Covid-19-related applications for funding and 11 were awarded to implement high-impact trials, pursue multicenter observational studies, support underserved patients with diabetes, and implement artificial intelligence and machine learning to predict respiratory failure in patients with Covid-19.

### New Technology and Innovation

The epidemic created many challenges that required innovative solutions. The DOM director of innovation (SPJ) led our efforts to use technology to overcome challenges the epidemic created. Some challenges that required innovative technological solutions included: **(1)** Converting most outpatient visits to video visits; this was achieved through several new platforms for video visits; **(2)** Connecting patients with their families as families were not allowed to visit; this was achieved by providing dozens of electronic tablets (donated to the department) to our inpatient floors and fitting them onto carts; **(3)** Rapid dissemination and updating of our treatment protocols; this was achieved by creating (with a developer) a free downloadable mobile App (MedProtocols, available on the iOS and Android App Stores) that stores all the Montefiore Covid-19 protocols.

### Recruiting Alumni Volunteers and Outreach for Donations

We contacted physician alumni of Einstein and Montefiore asking for volunteers to work in our hospitals during the pandemic. These efforts were highly successful with many alumni volunteering to come to the Bronx and work in our hospitals (Table 1). We also solicited donations through an Amazon page where we listed items needed (e.g., pulse oximeters for patients, etc). Through our newsletter and social media outreach we received numerous donations that not only helped us cope with shortages of these items but also gave a strong morale boost to our providers.

### Community Outreach and Efforts to Raise the Morale of Our Frontline Providers

Faced with a disease that had no treatment and caring for large numbers of patients decompensating and dying, the stress and heartbreak felt by our frontline providers cannot be overstated (17). We alleviated this stress through constant communication between leadership and the frontline providers (see above). We also collaborated with the Department of Psychiatry that established a strong mental health support program for the frontline providers at Montefiore. These measures were helpful, but we felt that we needed to do more. Therefore, we reached out to our Bronx, Westchester, and Hudson Valley communities through social media, and requested displays of support for the frontline providers at Montefiore. The response of the community was overwhelming and heartening. Hundreds of e-mails, letters, photos, videos, and artwork, mostly created by children, were sent to us and were shared with our frontline providers, energizing them and boosting their morale during these terrible times. We also launched a social media campaign under the hashtag **#MontefioreProud**. #MontefioreProud gear was distributed to DOM providers and staff and were highly popular and had a significant positive impact on morale and camaraderie at our institution.

## The Department of Medicine Post-COVID

As of the writing of this manuscript the positive Covid-19 testing rate has declined significantly in New York City and the epidemic seems to be contained. However, our work is not finished as we have to address two main remaining challenges: **(1)** preparing for additional Covid-19 surges; **(2)** treating patients who recovered from Covid-19 and continue to suffer from its long-term consequences.

### Preparing for Additional Surges

During the first Covid-19 wave at our institution we created an online database where we stored the information about all our deployed physicians. We have since updated this database with information about all physicians who may need to be deployed and where. Our approach is similar to military reserve preparedness where our central command can deploy physicians very quickly using this online database. Plans are in place for ICU expansion, IM units' expansion, and converting all our units into Covid-19 units, and rapid initiation of clinical trials.

### Treating Patients With Chronic Consequences of Covid-19

It is becoming evident that a large number of patients who survived Covid-19, especially those that were admitted to the ICU, suffer from long-term consequences of Covid-19 including chronic fatigue, dyspnea, foggy thinking, chronic anosmia, memory loss, depression, as well as cardiac, renal, pulmonary, and brain complications from the disease (18, 19). To address these issues the Montefiore DOM established a Covid Recovery (CORE) clinic where a multi-disciplinary team of clinicians are following patients who recovered from Covid-19. The CORE clinic will also serve as a research platform to study the long-term consequences of Covid-19, their causes, and how they can be prevented and treated.

## Lessons Learned

During a crisis such as the Covid-19 pandemic effective leadership can be described by the abbreviation **A,B,C,D,E**:

**Available** – the leadership team should always be available and responsive to all communications and requests coming from the frontlines.

**Back** – the providers at the frontlines should know and be constantly reassured that the leadership team always has their back and will support them with all resources available.

**Clear** – communications coming from the leadership team should be clear and consistent.

**Determined** – the leadership team should show determination and decisiveness, and decisions have to be made promptly without delay.

**Example** – the leadership team should lead by example.

The DOM leadership team, comprising the Medicine Covid-19 Taskforce (MCT) and our division chiefs adhered to these five principles: Our leadership team was available all the time and communicated with frontline providers continuously; we supported our frontline providers with all our resources, and when resources were insufficient (e.g., PPE) we solicited donations to acquire them; communications were clear, consistent, and timely; decisions were made promptly and unambiguously; and all members of our leadership team who did not have medical/age exemptions worked at the frontline together with our providers.

The first important lesson is that the 3C framework was effective, but only when applied at all levels of the organization and in a bi-directional way, from leadership to frontlines but also from frontlines to leadership (Figure 2).

One of the key components of the 3C framework was the establishment of the Medicine Covid-19 Taskforce (MCT). An important lesson learned is that the MCT had to be large to encompass all arms of the department. The MCT had 18 members and while that lengthened our meetings it was critical for coordinating such a large department.

The 3C framework enabled us not only to coordinate all arms of the DOM but also to quickly implement creative, out of the box, solutions to the new challenges we were facing such as switching inpatients consults to eConsults, or making all our protocols available through a free downloadable mobile App.

Finally, one of the most important lessons learned is that while the main focus was on deployment and expansion of our ICU's and IM units to accommodate the rapid inflow of Covid-19 patients, our work did not end with deployment only. We had to devote time and effort to research, education, outpatient care, donations, community outreach and much more, while at the same time expanding our capacity to care for Covid-19 inpatients. Here again the 3C framework was very helpful.

## Conclusions

The Covid-19 pandemic caused the most serious public health crisis of this century. No Medical Center in the US had experience with such a large-scale epidemic, and therefore hospitals had to adapt to the new situation and the many challenges it created overnight. Making things even worse, the lack of preparedness by government agencies for such a large-scale pandemic resulted in shortages of PPE, delayed testing for infection, and lack of clear and consistent treatment guidelines (20). Our medical center, located in the Bronx, was at the epicenter of the epidemic when it first hit the US during the months of March to May. In order to mobilize the DOM to handle the exponential increase in the number of patients with Covid-19 being admitted to our units we implemented a teamwork strategy that we dubbed the 3C framework. Using this framework, we were able to adapt quickly to the new situation in a coordinated and synergistic way, doubling our medicine units, deploying physicians, creating uniform and up-to-date treatment protocols, creating educational materials on treating Covid-19 for our frontline providers, converting inpatient consults and all outpatient activities into telemedicine, performing clinical trials, innovating and reaching out to our community through a large social media campaign. The main lesson from our experience is that the key to confronting such a challenging and stressful situation as the Covid-19 pandemic is to work in a cohesive leadership team that applies that 3C framework and functions in a coordinated and synergistic fashion. We believe that the same 3C framework can be effective not only within an individual Medical Center but when responding to a public health crisis at the regional and national level (3, 21, 22).

## Data Availability Statement

The original contributions presented in the study are included in the article/supplementary material, further inquiries can be directed to the corresponding author/s.

## Ethics Statement

Ethical review and approval were not required for the study since it did not involve human participants, in accordance with the local legislation and institutional requirements. Written informed consent was not required for the study since it did not involve human participants, in accordance with the national legislation and the institutional requirements.

## Author Contributions

YT: performed the work described in the manuscript for the Chair of Medicine, conceived the idea of the manuscript, and wrote the manuscript. MN: performed the work described in the manuscript for the Chief of Critical Care Medicine and wrote certain sections of the manuscript. MK: performed the work described in the manuscript for the Vice Chair of Medicine for Research and wrote certain sections of the manuscript. WS: performed the work described in the manuscript for the Chief of Hospital Medicine and wrote certain sections of the manuscript. EK: performed the work described in the manuscript for the Vice Chair for Faculty Affairs and wrote certain sections of the manuscript. GK: performed the work described in the manuscript for the Residency Program Director (Wakefield campus) and wrote certain sections of the manuscript. LS: performed the work described in the manuscript for the Residency Program Director (Moses-Weiler campuses) and wrote certain sections of the manuscript. SJ: performed the work described in the manuscript for the Department of Medicine Director of Innovation and wrote certain sections of the manuscript. EE: performed the work described in the manuscript for the Director of Medicine Faculty Practice and wrote certain sections of the manuscript. All authors contributed to the article and approved the submitted version.

## Conflict of Interest

YT was previously (1/2015 – 6/2017) the PI on a basic research project jointly funded by the Juvenile Diabetes Research Foundation and Pfizer that is unrelated to the current manuscript. YT has two patent applications. (PCT/US2016/067775 & US provisional No: 61/883,062) that are not related to the current manuscript. The remaining authors declare that the research was conducted in the absence of any commercial or financial relationships that could be construed as a potential conflict of interest.

## References

[1] Zangrillo A Beretta L Silvani P Colombo S Scandroglio AM Dell'Acqua A. Fast reshaping of intensive care unit facilities in a large metropolitan hospital in Milan, Italy: facing the COVID-19 pandemic emergency. Crit Care Resusc. (2020) 22:91–4. 32227819 10.51893/2020.2.pov1 PMC10692483

[2] Ciceri F Castagna A Rovere-Querini P De Cobelli F Ruggeri A Galli L. Early predictors of clinical outcomes of COVID-19 outbreak in Milan, Italy. Clin Immunol. (2020) 217:108509. 10.1016/j.clim.2020.108509 32535188 PMC7289745

[3] Emanuel EJ Persad G Upshur R Thome B Parker M Glickman A. Fair allocation of scarce medical resources in the time of covid-19. N Engl J Med. (2020) 382:2049–55. 10.1056/NEJMsb2005114 32202722

[4] Keller MJ Kitsis EA Arora S Chen JT Agarwal S Ross MJ. Effect of systemic glucocorticoids on mortality or mechanical ventilation in patients with covid-19. J Hosp Med. (2020) 15:489–93. 10.12788/jhm.3497 32804611 PMC7518134

[5] Wexler DJ Hirsch IB Mulder JE. Coronavirus disease 2019 (COVID-19): issues related to diabetes mellitus in adults. UpToDate. (2020).

[6] Richterman A Meyerowitz EA Cevik M. Hospital-acquired SARS-CoV-2 infection: lessons for public health. JAMA. (2020). 10.1001/jama.2020.21399. [Epub ahead of print]. 33185657

[7] Spitzer Sverd S Gardner LE Cabassa JA Schneider M Noone RH Jahdi MH. A Bronx tale: exposure, containment and care on inpatient psychiatry units during COVID-19. Gen Hosp Psychiatry. (2020). 10.1016/j.genhosppsych.2020.07.010. [Epub ahead of print]. 32917397 PMC7399657

[8] Merken R Kruger A Bhardwaj G Kajita GR Shapiro LI. Internal medicine resident work absence during the COVID-19 pandemic at a large academic Medical Center in New York City. J Grad Med Educ. (2020) 12:682–5. 10.4300/JGME-D-20-00657.1 33391591 PMC7771614

[9] Bernstein CA Bhattacharyya S Adler S Alpert JE. Staff emotional support at montefiore Medical Center during the covid-19 pandemic. Jt Comm J Qual Patient Saf. (2020) 47:185–9. 10.1016/j.jcjq.2020.11.009 33353851 PMC7673209

[10] Agarwal S Schechter C Southern W Crandall JP Tomer Y. Preadmission diabetes-specific risk factors for mortality in hospitalized patients with diabetes and coronavirus disease 2019. Diabetes Care. (2020) 43:2339–44. 10.2337/dc20-1543 32769128 PMC7510015

[11] Reyes Gil M Barouqa M Szymanski J Gonzalez-Lugo JD Rahman S Billett HH. Assessment of lupus anticoagulant positivity in patients with coronavirus disease 2019 (COVID-19). JAMA Netw Open. (2020) 3:e2017539. 10.1001/jamanetworkopen.2020.17539 32785632 PMC12124689

[12] Palaiodimos L Kokkinidis DG Li W Karamanis D Ognibene J Arora S. Severe obesity, increasing age and male sex are independently associated with worse in-hospital outcomes, and higher in-hospital mortality, in a cohort of patients with COVID-19 in the Bronx, New York. Metabolism. (2020) 108:154262. 10.1016/j.metabol.2020.154262 32422233 PMC7228874

[13] Fisher M Neugarten J Bellin E Yunes M Stahl L Johns TS. AKI in hospitalized patients with and without covid-19: a comparison study. J Am Soc Nephrol. (2020) 31:2145–57. 10.1681/ASN.2020040509 32669322 PMC7461660

[14] Nori P Cowman K Chen V Bartash R Szymczak W Madaline T. Bacterial and fungal coinfections in COVID-19 patients hospitalized during the New York City pandemic surge. Infect Control Hosp Epidemiol. (2020) 42:1–5. 10.1017/ice.2020.368 32703320 PMC7417979

[15] Chand S Kapoor S Orsi D Fazzari MJ Tanner TG Umeh GC. COVID-19-associated critical illness-report of the first 300 patients admitted to intensive care units at a New York city Medical Center. J Intensive Care Med. (2020) 35:963–70. 10.1177/0885066620946692 32812834

[16] Sourial MY Sourial MH Dalsan R Graham J Ross M Chen W. Urgent peritoneal dialysis in patients with covid-19 and acute kidney injury: a single-center experience in a time of crisis in the United States. Am J Kidney Dis. (2020) 76:401–6. 10.1053/j.ajkd.2020.06.001 32534129 PMC7287441

[17] Cunningham CO Diaz C Slawek DE. COVID-19: the worst days of our careers. Ann Intern Med. (2020) 172:764–5. 10.7326/M20-1715 32282870 PMC7161307

[18] Carfi A Bernabei R Landi F Gemelli against C-P-ACSG. Persistent symptoms in patients after acute COVID-19. JAMA. (2020) 324:603–5. 10.1001/jama.2020.12603 32644129 PMC7349096

[19] Li Z Zheng C Duan C Zhang Y Li Q Dou Z. Rehabilitation needs of the first cohort of post-acute COVID-19 patients in Hubei, China. Eur J Phys Rehabil Med. (2020) 56:339–44. 10.23736/S1973-9087.20.06298-X 32672029

[20] Shapiro LI Kajita GR Arnsten JH Tomer Y. Toward better preparedness for the next pandemic. J Clin Invest. (2020) 130:4543–45. 10.1172/JCI140296 32574154 PMC7456246

[21] Schuchat A Team CC-R. Public Health Response to the Initiation and Spread of Pandemic COVID-19 in the United States, February 24–April 21, 2020. MMWR Morb Mortal Wkly Rep. (2020) 69:551–6. 10.15585/mmwr.mm6918e2 32379733 PMC7737947

[22] Haffajee RL Mello MM. Thinking Globally, Acting Locally—The U.S. Response to Covid-19. N Engl J Med. (2020) 382:e75. 10.1056/NEJMp2006740 32240580

